# Emerging threats of H5N1 clade 2.3.4.4b: cross-species transmission, pathogenesis, and pandemic risk

**DOI:** 10.3389/fcimb.2025.1625665

**Published:** 2025-07-25

**Authors:** Riaz-M Azeem, Ying-Shi Yang, Siddique Sehrish, Chun-Wei Shi, Gui-Lian Yang, Suthar-Teerath Kumar, Wen-Tao Yang, Chun-Feng Wang

**Affiliations:** ^1^ College of Veterinary Medicine, Jilin Provincial Engineering Research Center of Animal Probiotics, Jilin Provincial Key Laboratory of Animal Microecology and Healthy Breeding, Jilin Agricultural University, Changchun, China; ^2^ Engineering Research Center of Microecological Vaccines (Drugs) for Major Animal Diseases, Ministry of Education, Jilin Agricultural University, Changchun, China

**Keywords:** cattle, cross-species barrier, HPAIV H5N1, mammals, molecular adaptations, reassortment

## Abstract

H5N1 is a highly pathogenic avian influenza virus (HPAIV) with zoonotic potential, posing a significant risk to animal health, agriculture, and human public health. A particular strain, clade 2.3.4.4b, has spread globally and has been detected in various mammalian species—including cattle and a limited number of human cases—highlighting its potential to spark a pandemic. Investigating this specific clade represents a crucial step toward the development of effective preventive and therapeutic strategies. This mini-review aims to outline the etiology and pathophysiological mechanisms driving the current bird flu outbreak in cattle. A targeted literature search was conducted in PubMed for studies published between 2003 and 2025 using keywords such as “bird flu”, “cattle”, “mammals”, “H5N1”, and “pathogenesis”. This review explores the pathogenic mechanisms and clinical manifestations associated with HPAIV H5N1 infections in mammals specially in cattle. A key hypothesis is that the ongoing outbreak is fueled by molecular adaptations in the virus that enhance its ability to cross species barriers. As these mechanisms continue to be uncovered, there is a pressing need for high-quality research to inform pandemic preparedness, guide effective control strategies, and support the development of targeted vaccines and antiviral therapies.

## Introduction

H5N1 is a highly pathogenic avian influenza virus (HPAIV) with zoonotic potential, posing a significant risk to animal health, agriculture, and human public health (FAO, 2025). Although avian influenza viruses primarily infect wild birds, they have demonstrated the ability to cross species barriers, infecting a wide range of hosts including terrestrial and marine mammals. Traditionally, mammals were not considered frequent hosts of HPAIV. However, since March 2024, clade 2.3.4.4b of H5N1 has been detected in cattle and has also resulted in human infections, raising significant concern within the scientific community (Pardo-Roa et al., 2025). According to the most recent data from the U.S. Centers for Disease Control and Prevention (CDC), the virus has spread to cattle in 17 states, affecting 1,007 dairy herds (CDC, 2025). This recent outbreak is believed to be driven by molecular adaptations that enhance the virus’s capacity for interspecies transmission (Pardo-Roa et al., 2025; Song et al., 2025). Genetic analyses further indicate that novel variants have emerged through reassortment events between low-pathogenic and highly pathogenic strains circulating in wild avian fauna (Sun et al., 2025). The pathogenesis of avian influenza is complex, involving dynamic interactions between viral and host factors. From an epidemiological standpoint, the global distribution and impact of avian influenza remain highly concerning. Given the rapid global spread of clade 2.3.4.4b, its repeated spillover into mammalian hosts, and the unpredictability of its genetic evolution, this variant represents a serious pandemic threat.

## Diagnostic criteria for H5N1 in mammals

Influenza is typically diagnosed based on a combination of clinical presentation and epidemiological risk factors. In humans, the initial symptoms of H5N1 infection are often nonspecific and may include eye redness, anorexia, myalgia (muscle pain), headache, fever, chills, or shivering. These symptoms usually have a sudden onset and are commonly accompanied by respiratory issues such as a dry cough, sore or dry throat (sometimes with hoarseness), and nasal congestion or discharge. Coughing is the most prevalent respiratory symptom and may be accompanied by substernal burning or pain. In elderly individuals and those with compromised immune systems, the initial presentation may be less pronounced due to a diminished cytokine response. However, these patients are still at risk of progressing to severe lower respiratory tract disease. Additional symptoms such as fever, fatigue, and confusion may occur even in the absence of pronounced respiratory involvement. In children, febrile seizures can sometimes be the initial symptom, even when other systemic signs are mild or absent (Krammer et al., 2018; WHO, 2025).

**Figure 1:**
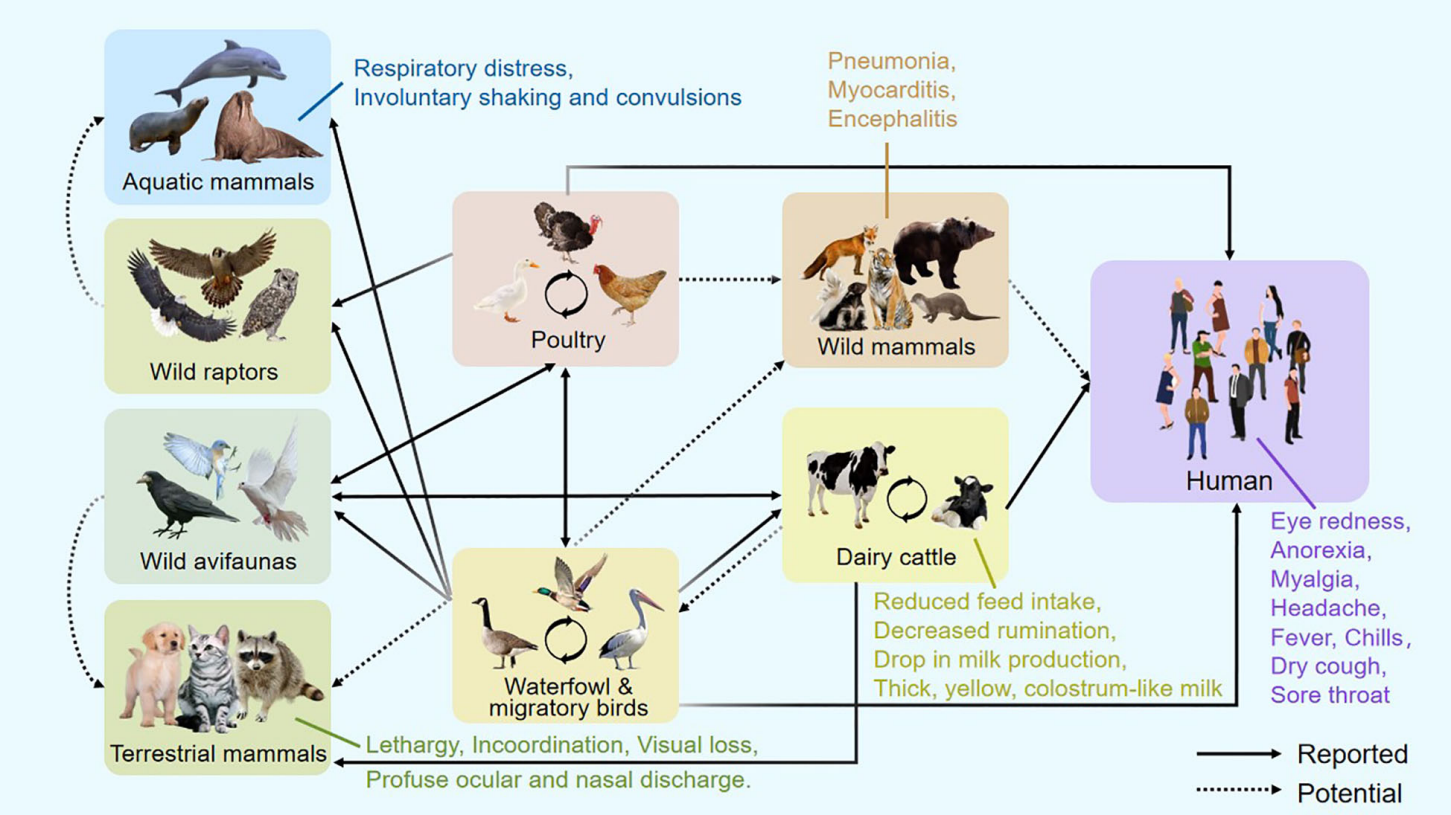
Host range and clinical manifestations of H5N1 bird flu.

Experimental infection of ferrets with the human-isolated Chile/25945 A(H5N1) virus produced clinical signs including fever, nasal discharge, diarrhea, and ocular discharge, along with increased lethargy and persistent signs of severe illness such as labored breathing (Pulit-Penaloza et al., 2024). Amid the recent surge of H5N1 infections in American dairy herds, humans in direct contact with infected animals exhibited relatively mild symptoms, such as fatigue, conjunctivitis, and eye redness due to inflammation (**Figure 1**) (AVMA, 2025). Because clinical features are often nonspecific, bird flu infection in humans cannot be confirmed through symptoms alone; laboratory testing is essential. Diagnosis typically involves collecting a nasal or throat swab for molecular testing, with the highest accuracy achieved when samples are obtained early in the course of illness.

Infected dairy cattle during the same outbreak displayed nonspecific clinical signs, including reduced feed intake, decreased rumination, and a sudden drop in milk production (**Figure 1**). The milk appeared thick, yellow, and colostrum-like. Clade 2.3.4.4b H5N1 was detected in the milk and found to cause mastitis through replication in mammary alveolar epithelial cells (Burrough et al., 2024). Symptoms in cows typically peaked within 4–6 days and resolved over 10–14 days, after which milk production normalized. Farm cats that consumed infected milk developed severe neurological symptoms, including lethargy, rigid movements, incoordination, visual loss, circling behavior, and profuse ocular and nasal discharge. Neurological assessments revealed absent menace responses and pupillary light reflexes, along with a diminished blink reflex (Burrough et al., 2024).

Evidence of H5N1 neurotropism has also been documented in other mammals. In wild red foxes (*Vulpes vulpes*), the virus has been associated with neurological involvement (**Figure 1**) (Bordes et al., 2023). Similarly, in bottlenose dolphins (*Tursiops truncatus*), pathological findings revealed neuronal necrosis and inflammation of the brain and meninges, with the highest viral loads detected in brain tissue (Murawski et al., 2024). Infected U.S. harbor seals and Peruvian sea lions displayed respiratory distress (including labored breathing and nasal secretions) alongside neurological manifestations such as involuntary shaking and convulsions (Gamarra-Toledo et al., 2023). These findings suggest that, as in birds, H5N1 exhibits a marked neurotropism in mammals, often leading to serious illnesses and pathological changes such as encephalitis. Therefore, neural tissue should be emphasized in wildlife monitoring efforts to ensure precise identification of H5N1 infections in mammalian species (Vreman et al., 2023).

## Susceptibility to H5N1

The susceptibility of the HPAIV H5N1 varies significantly across host species, shaped by multifaceted dynamics among viral genetics, host biology, and surrounding ecological factors. Initially adapted to avian hosts, H5N1 continues to circulate endemically among wild aquatic birds—particularly migratory waterfowl such as ducks and geese—which typically remain asymptomatic or exhibit only mild symptoms. These birds act as efficient carriers, disseminating the virus across long distances without manifesting clinical disease, thereby posing a continual threat to domestic poultry (Giacinti et al., 2024; Mao et al., 2024). In contrast, domestic birds such as chickens and turkeys are highly vulnerable, often developing rapidly progressing systemic disease with exceptionally high mortality rates (Cha et al., 2025).

Beyond birds, H5N1 has demonstrated an increasing capacity to infect a wide range of mammalian species, including cattle, humans, pigs, dogs, cats, tigers, and marine mammals like sea lions [**Figure 1** modified from article (Butt et al., 2024)] (Abdelwhab and Mettenleiter, 2023; Ulloa et al., 2023; Burrough et al., 2024). While animals such as horses, pigs, dogs, poultry, and wild birds have long been recognized as reservoir hosts of influenza A viruses (IAV), recent infections in non-traditional hosts—including marine mammals, minks, cattle, raccoons, foxes, and domestic cats—highlight an expanding host range not typically associated with IAV maintenance (Peacock et al., 2025). Research suggests that the current bovine outbreak of H5N1 likely originated from wild birds or poultry, followed by cow-to-cow transmission via contaminated milking equipment such as teat cups and other fomites (Cohen, 2024; Guan et al., 2024; Le Sage et al., 2024; Nguyen et al., 2025). Additionally, mechanical vectors such as house flies and aerosol transmission may contribute to the spread of the virus within cattle populations (Baker et al., 2024). These cross-species transmissions reflect the virus’s ongoing adaptation and underscore its potential for zoonotic and pandemic escalation.

Species-specific differences in disease outcomes are well established. Ducks often carry the virus subclinically, whereas chickens and turkeys develop acute, high-fatality infections with widespread tissue involvement. In humans, H5N1 infection is rare but severe, with a case fatality rate estimated at around 50% (WHO, 2025). Most human infections result from direct or indirect exposure to infected poultry, ducks, or cattle, rather than through sustained human-to-human transmission (CDC, 2025; Lukarska et al., 2017). However, the recent detection of H5N1 in unconventional mammalian hosts, including cattle and pinnipeds, suggests the virus is adapting to new species, potentially expanding its ecological niche and complicating outbreak control.

Several factors influence susceptibility across species. On the viral side, mutations in the hemagglutinin (HA) cleavage site and polymerase basic protein 2 (PB2) have been shown to enhance replication in mammalian cells and facilitate interspecies transmission (Halwe et al., 2025; Pardo-Roa et al., 2025; Yang et al., 2025). On the host side, the localization of sialic acid receptors plays a critical role. Avian species primarily express α2,3-linked sialic acid receptors in their respiratory and gastrointestinal tracts, which are preferentially targeted by H5N1. Humans, in contrast, predominantly express α2,6-linked receptors in the upper respiratory tract, though α2,3-linked receptors are present in the lower airways, providing a limited entry route for avian viruses. Notably, clade 2.3.4.4b H5N1 strains isolated from cattle exhibit dual receptor-binding capacity, allowing interaction with both α2,3 (avian-type) and α2,6 (human-type) receptors, raising concerns about their ability to infect human upper respiratory tract cells (Eisfeld et al., 2024; Song et al., 2025). Alarmingly, Lin et al. demonstrated that a single mutation in the HA protein of bovine-derived H5N1 can switch receptor binding preference toward human receptors—an evolutionary step with significant public health implications (Lin et al., 2024).

Host immune status, including prior exposure or vaccination, also modulates susceptibility, influencing both infection likelihood and disease severity (Wu et al., 2019). Moreover, environmental and geographic factors strongly shape transmission dynamics. Regions with intensive poultry farming, live animal markets, and poor biosecurity create ideal conditions for viral amplification, reassortment, and interspecies transmission. Additionally, migratory bird movements link distant ecosystems and serve as a major conduit for the transcontinental spread of H5N1 strains (Caliendo et al., 2022).

## Pathogenesis of H5N1

Numerous viral determinants contributing to the virulence and pathogenesis of HPAIV H5N1 have been identified through genetic manipulation and studies using mammalian models. Among these, the hemagglutinin (HA), neuraminidase (NA), and the viral RNA polymerase complex (comprising PB1, PB2, and PA proteins) play central roles. The hemagglutinin (HA) protein of HPAIV H5N1 displays an elevated pH threshold for membrane fusion relative to that of conventional avian influenza strains. Although this feature renders HA more vulnerable to inactivation within the avian gut or external environments, it facilitates efficient host cell entry in mammals, thereby broadening the virus’s host range despite the endosomal pH barrier (Daidoji et al., 2017). A critical factor underlying the high pathogenicity of certain H5 and H7 strains is the presence of multibasic cleavage sites (MBCS) within the HA protein. These MBCS are efficiently cleaved by host proteases such as PC6 and furin, enabling systemic viral spread and severe disease (Izaguirre, 2019).

Recent H5N1 outbreaks in cattle have highlighted notable changes in HA, particularly slight shifts in binding affinity toward human-like α2,6-linked sialic acid receptors (Lin et al., 2024; Song et al., 2025). While some studies report that bovine-derived H5N1 HA proteins still retain the receptor-binding and fusion characteristics typical of avian-adapted viruses (Yang et al., 2025), a group of researchers have demonstrated that a point mutation in bovine-origin HA can shift receptor specificity toward human-type receptors—a potentially critical step toward zoonotic adaptation.

The M1 matrix protein also contributes to virulence. Mutations in the M1 gene, including those promoting a filamentous viral morphology and post-translational SUMOylation, have been shown to enhance viral replication and pathogenicity in mammalian hosts (Guo et al., 2022). Additionally, the non-structural protein NS1 plays a key role in immune evasion by antagonizing host antiviral responses. Specific amino acid residues and regions within NS1 are directly linked to enhanced virulence through inhibition of interferon signaling and other immune pathways (Mok et al., 2017; Tseng et al., 2021).

During the recent bovine outbreaks, the PB2 protein emerged as a critical factor in viral adaptation. Bovine H5N1 isolates have exhibited the E627K mutation in PB2—a well-characterized change known to enhance polymerase activity and replication efficiency in mammalian cells (Halwe et al., 2025; Nguyen et al., 2025; Song et al., 2025). Both PB1 and PB2 contribute to immune modulation: PB1 interferes with mitochondrial antiviral signaling (MAVS), while PB2 suppresses the JAK1/STAT pathway, dampening host immune defenses (Leymarie et al., 2017; Yang et al., 2021). Notably, three mutations—PB1 E177G, A652T, and NP I119M—were identified in the central nervous system of a ferret that developed severe meningoencephalitis after infection with H5N1 (A/Indonesia/5/2005), underscoring their role in neurovirulence (Siegers et al., 2023).

The PA protein, another component of the polymerase complex, is also implicated in H5N1 severity across multiple host species (Nogales et al., 2021). Together, these viral proteins and mutations illustrate the multifactorial nature of H5N1 pathogenesis and underscore the virus’s ongoing adaptation to mammalian hosts.

## H5N1 dysregulating the immune system

**Figure 2:**
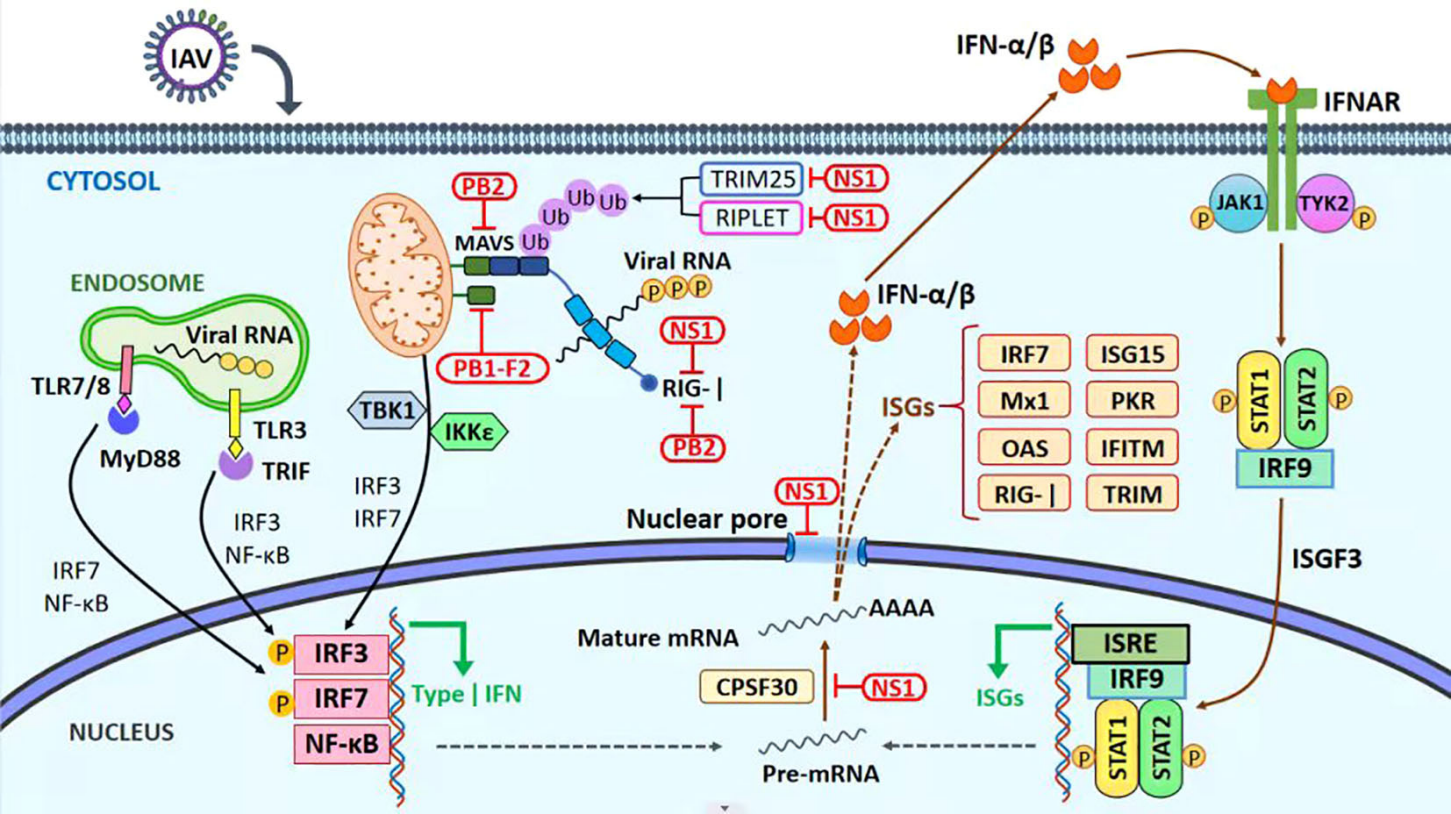
Immune evasion mechanism. The antiviral innate immune signaling pathways and viral immune evasion strategies during influenza A virus infection. RIG-I is one of the host-pathogen recognition receptors (PRRs) that recognize 50-triphosphate single-stranded RNA (ssRNA) and double-stranded RNA (dsRNA) products produced by viral replication. As a result, RIG-I undergoes a conformational shift that exposes its TRIM25-ubiquitinated CARD domains as a result of RIG-I’s subsequent binding to MAVS at the mitochondrial membrane, a subsequent signaling cascade that includes the triggering of the transcription factors IRF3, IRF7, and NF-kB results in the generation of type-I IFN. Infected cells with the IAV secrete IFN-I, which binds to IFNAR receptors on the cell surface and causes JAK1/TYK2 to be phosphorylated. Next, STAT1 and STAT2 are bound and phosphorylated, causing them to join with IRF9 to form a complex. Hundreds of genes, including ISGs, will be activated and transcribed by the ISGF-3 complex, which will operate as a transcription factor. ISGs play a crucial role in moderating the host antiviral mechanism. Red boxes indicate regions where IAV proteins interact or restrict the host’s innate immune response.

The most corrival of the host innate immunologic retaliation during viral replication is regarded to be the NS1 of the AIV (Mok et al., 2017; Tseng et al., 2021). NS1 targets the RNA sensing-TRAF-3 type I IFN axis by resembling a TRAF-3 interacting motif and impairs innate antiviral immunity (Lin et al., 2021). NS1 also interacts with RIG-1 and controls the innate immune response in many ways (Yang et al., 2021; Wang et al., 2022). The suppressor of kappa B kinase (IKK) is also inhibited by NS1, which consequently disrupts the nuclear factor kappa B (NF-κB) signaling cascade, thereby preventing the activation of numerous antiviral genes [**Figure 2**. modified from the article (Muñoz-Moreno et al., 2021)] (Gao et al., 2012).

The host protein calcium binding and coiled-coil domain 2 (NDP52) of the PB1-F2 modulate the MAVS, inhibiting the host immune IFN response (Varga et al., 2011; Leymarie et al., 2017). The PB2 protein of the influenza virus escapes the innate immune response of host cells by inhibiting the JAK1/STAT signaling (Yang et al., 2022). M2 protein also plays its role in suppressing the innate immune response. M2 inhibits RLR signaling and autophagic antiviral response via the activation of MAVS signaling [reviewed in (Muñoz-Moreno et al., 2021)].

The PA protein is the mainstream outcome translated from IAV RNA segment 3. Another open reading frame (ORF), PA-X, was identified to create a highly preserved fusion protein among different strains (Jagger et al., 2012). PA-X uses the host RNAs Xrn1 to split the host transcripts in the nucleus and help the processing of IAV mRNAs (Khaperskyy et al., 2016). PA-X protein of the HPAIV H5N1 restricts NF-κB activity, which is a possible mechanism for PA-X thwarting host immune responses (Hu et al., 2015).

The AIV polymerase also contributes to managing the host antiviral response. It must be remembered that some AIV proteins and viral polymerase, too, have their role in virulence and pathogenicity. So, it is not shocking that viruses having well-ordered polymerases have better chances of evading the host immune antiviral responses (Grimm et al., 2007). The multifaceted pathogenesis of HPAIV H5N1 includes viral replication and disruption of cytokines and chemokines; immune factor dysregulation, including elevated levels of TNF-related apoptosis-inducing ligand (TRAIL) and reduced cytotoxicity of CD8+ cells, has been linked to the disease’s progression (Gobbo et al., 2022; Dey et al., 2023). The pathogenesis of HPAIV H5N1 may be strongly impacted by the activation of functional TRAIL, an inducer of cell apoptosis, in virus-infected macrophages (Roberts, 2023).

## H5N1 vaccine development

Although the complete eradication of H5N1 from its natural reservoirs is not currently feasible, proactive preparation for a potential future pandemic caused by an H5N1 derivative remains essential. Notably, some viral isolates within clade 2.3.4.4b have developed mutations that confer resistance to antiviral drugs by altering drug-binding sites (Zhang et al., 2025). To mitigate the risk of resistance-driven viral escape and reassortment, it is advisable to pursue multi-target antiviral regimens that reduce the likelihood of selecting for drug-resistant variants.

Encouragingly, several experimental vaccine candidates have shown promising results in preclinical studies. These vaccines have elicited strong neutralizing antibody responses, mucosal IgA production, and robust T cell immunity in animal models, particularly mice (Liu et al., 2025). Moreover, vaccination programs in ducks have proven effective in limiting the spread of highly pathogenic H5N1, helping to reduce broader panzootic risks (Guinat et al., 2025). An influenza mRNA vaccine has also demonstrated protective efficacy in ferrets, preventing lethal infection from HPAIV H5N1 (Hatta et al., 2024). Vaccine stockpiles targeting H5 viruses that are antigenically similar to the currently circulating clade 2.3.4.4b strains are available. If H5N1 begins to spread among humans, these vaccines could be quickly produced in large quantities using mRNA technology (Furey et al., 2024).

Interestingly, a recent study evaluating seasonal influenza vaccines found that they could boost antibody titers to protective levels against H5N1, suggesting that such vaccines might serve as an initial defense strategy in the early phase of an H5N1 outbreak, pending deployment of strain-specific pandemic vaccines (Sanz-Muñoz et al., 2025). Additionally, intranasal administration of a DelNS1-LAIV (live attenuated influenza vaccine) platform has shown promising results, providing durable protection against lethal H5N1 challenge in both female mice and male hamster models, including those infected with cattle- and mink-adapted variants (Liu et al., 2025).

Ongoing research continues to advance H5N1 vaccine development, with the goal of creating broadly protective, rapidly deployable immunization strategies to safeguard against emerging variants and future zoonotic threats.

## Future prospects

The ongoing genetic evolution of H5N1, particularly within clade 2.3.4.4b, has resulted in greater viral diversity, with some strains exhibiting enhanced adaptation to mammalian hosts. Such adaptations elevate concerns about the virus’s pandemic potential, especially given the possibility of reassortment with seasonal human influenza viruses. A successful reassortant could combine the virulence of H5N1 with efficient human-to-human transmissibility, drastically altering the global susceptibility landscape. The current adaptations and reassortments have caused outbreak in cattle affecting 17 different states and more than 1000 dairy herds.

Although continuous transmission between humans has not been observed to date, the continued zoonotic threat posed by H5N1 necessitates vigilant surveillance and proactive intervention. Populations with occupational exposure to poultry and wild birds, such as farmers, cullers, and veterinarians, remain at heightened risk. The progressive expansion of H5N1’s host range, coupled with its high lethality and genetic plasticity, underscores the critical need for enhanced global surveillance systems, rigorous biosecurity measures, and the development of broadly protective vaccines and antiviral strategies aimed at mitigating the risks associated with this formidable pathogen.

The prevention measures to mitigate the effects of H5N1 are necessary because it also poses significant threat to food security. We have seen the significant shortage of eggs in the United States. Furthermore, it also risks the milk and meat production. These outcomes from outbreak also pose concernig damages economically. Given the scale of the ongoing H5N1 panzootic, continuous and coordinated surveillance is critical to detect any escalating threats to biodiversity and human health. It is crucial for all impacted nations to openly disclose complete and detailed information—including viral genomic sequences, affected species, and case numbers. Prompt and open communication of findings is crucial. Strengthening international collaboration is imperative to ensure rapid response efforts, particularly in under-resourced regions where technological and logistical limitations may hinder timely data generation and analysis. Support for these regions is necessary to improve global preparedness and mitigate the risk of H5N1 spillover into mammals, which could potentially trigger a wider panzootic or human pandemic.

Furthermore, it is vital to reassess the human–animal–environment interface to reduce the emergence of high-risk pathogens. Governments must take proactive charge for safeguarding species richness and public health, especially against ailments driven by human activity—such as those linked to intensive farming practices, including poultry production. Emerging technologies like mRNA-based vaccines, advanced genomic sequencing, and CRISPR–Cas diagnostic tools offer swift and adaptable solutions for managing outbreaks, but their effectiveness is limited if their deployment is restricted on-site at farms. To effectively prevent future outbreaks, preserve ecosystems, and protect global health, we must rethink our food production systems and minimize our ecological disruption, particularly in our interactions with wildlife.
